# Molecular Fingerprint of Cold Adaptation in Antarctic Icefish PepT1 (*Chionodraco hamatus*): A Comparative Molecular Dynamics Study

**DOI:** 10.3390/biom15081058

**Published:** 2025-07-22

**Authors:** Guillermo Carrasco-Faus, Valeria Márquez-Miranda, Ignacio Diaz-Franulic

**Affiliations:** Center for Bioinformatics and Integrative Biology, Universidad Andres Bello, Santiago 8370146, Chile

**Keywords:** cold adaptation, membrane transporter dynamics, molecular simulations

## Abstract

Cold environments challenge the structural and functional integrity of membrane proteins, requiring specialized adaptations to maintain activity under low thermal energy. Here, we investigate the molecular basis of cold tolerance in the peptide transporter PepT1 from the Antarctic icefish (*Chionodraco hamatus*, ChPepT1) using molecular dynamics simulations, binding free energy calculations (MM/GBSA), and dynamic network analysis. We compare ChPepT1 to its human ortholog (hPepT1), a non-cold-adapted variant, to reveal key features enabling psychrophilic function. Our simulations show that ChPepT1 displays enhanced global flexibility, particularly in domains adjacent to the substrate-binding site and the C-terminal domain (CTD). While hPepT1 loses substrate binding affinity as temperature increases, ChPepT1 maintains stable peptide interactions across a broad thermal range. This thermodynamic buffering results from temperature-sensitive rearrangement of hydrogen bond networks and more dynamic lipid interactions. Importantly, we identify a temperature-responsive segment (TRS, residues 660–670) within the proximal CTD that undergoes an α-helix to coil transition, modulating long-range coupling with transmembrane helices. Dynamic cross-correlation analyses further suggest that ChPepT1, unlike hPepT1, reorganizes its interdomain communication in response to temperature shifts. Our findings suggest that cold tolerance in ChPepT1 arises from a combination of structural flexibility, resilient substrate binding, and temperature-sensitive interdomain dynamics. These results provide new mechanistic insight into thermal adaptation in membrane transporters and offer a framework for engineering proteins with enhanced functionality in extreme environments.

## 1. Introduction

Temperature has shaped protein evolution, crafting the molecular tools that underpin species survival in extreme environments [1,2]. In polar environments, organisms sustain metabolism despite limited thermal energy, relying on cold-adapted proteins with enhanced structural flexibility [3,4]. This flexibility, driven by strategic mutations that weaken intramolecular interactions at key hinge regions, lowers energy barriers for catalysis and transport—offering insights into how proteins evolve to overcome kinetic constraints in extreme cold [5,6,7]. The cold tolerance of membrane proteins has been afforded mostly from a “lipid-centric” perspective, focusing on their increased viscosity at low temperatures [8,9,10]. This rigidification, caused by tighter lipid packing at low temperatures, restricts protein mobility and highlights the role of homeoviscous adaptation in preserving their function. However, there are a few examples where additional phenomena need to be considered for a thorough explanation of cold tolerance. The temperature sensitivity of the cardiac Na^+^/Ca^2+^ exchanger is largely determined by sequence differences within the NH_2_-terminal transmembrane segments, with the first four transmembrane regions accounting for most of the differences between trout and canine isoforms, although no specific residues were identified as responsible for cold tolerance [11]. *Rana sylvatica* SERCA1 shows cold adaptations with higher near-freezing activity, reduced activation energy (E_a_), and unique Michaelis-Menten kinetics. Seven amino acid substitutions, especially in the ATP-binding domain, enhance function, though their precise mechanism remains unclear [12]. The Antarctic Na^+^/K^+^-ATPase is an intrinsically cold-tolerant membrane transporter [13]. Interestingly, Galarza-Muñoz et al. found that a single residue (L314) accounts for the cold-tolerant phenotype and, moreover, that a hydrophobic accessible surface area (hASA) cutoff at this position determines the height of the energy barrier for ion transport revealing a novel mechanism of cold adaptation [14]. The peptide/proton co-transporter 1 (PepT1) is a proton-coupled oligopeptide transporter found in the intestinal epithelium of vertebrates [15] that exhibits a remarkable cold tolerance in the Antarctic icefish (*Chionodraco hamatus*) [16]. Previous research identified a 6x heptad repeat motif (VDMSRKS) in the C-terminal domain (CTD) of *Chionodraco hamatus* PepT1 (ChPepT1) as a critical element for its thermal tolerance. Interestingly, a single heptad is required to confer full cold tolerance to chPepT1, but its removal still fails to match the levels observed in the temperate PepT1 (rabbit), suggesting the existence of additional, yet unknown, factors [16]. Here, we used the structure of the human hPepT1 (hPepT1) (PDB ID: 7PMW [17]) and the structural model of the *Chinondraco hamatus* PepT1 (ChPepT1) available in the AlphaFold database in the occluded outward-facing conformation and performed molecular simulations of both temperate and cold-tolerant variants at 273 K and 323 K. Our results reveal that the Antarctic PepT1 is intrinsically more flexible than its temperate counterpart, consistent with patterns observed in other cold-adapted enzymes. Relative binding free energy calculations show that while hPepT1 loses binding affinity for the dipeptide as temperature increases, ChPepT1 maintains stable substrate interactions across the full temperature range. Additionally, ChPepT1 exhibits temperature-sensitive remodeling of its interactions with membrane lipids, particularly near the substrate-binding site. Notably, the proximal carboxy-terminal domain (CTD) of ChPepT1 undergoes a conformational transition from random coil to α-helix at low-temperature and remains closely associated with key transmembrane helices. Correlated motion analyses further reveal that CTD dynamics are linked to the peptide-binding site, suggesting a role for this domain in stabilizing the transport pathway under cold conditions. These findings propose a previously unrecognized mechanism by which CTD contributes to cold tolerance, offering broader insights into how membrane transport proteins can adapt structurally and functionally to extreme environments.

## 2. Materials and Methods

The peptide transporter 1 (PepT1) structure from *Homo sapiens* in the outward facing occluded conformation bound to an Ala-Phe dipeptide was obtained from the Protein Data Bank (PDB ID:7PMW), solved by cryo-EM at a 4.10 Å resolution, while PepT1 structure from *Chionodraco hamatus* (PepT1-CH) was obtained from the AlphaFold Protein Structure Database (ID:Q804I3), average pLLDT of 85.31. An Ala-Phe dipeptide was placed in the PepT1-CH structure in the same location as found in the human PepT1 structure. The proteins and their corresponding dipeptides were incorporated into a palmitoyl-oleoyl-phosphatidylcholine (POPC) membrane bilayer utilizing CHARMM-GUI [18]. The membrane-integrated assembly was immersed in a TIP3P aqueous environment containing 400 mM NaCl salt concentration, mimicking the natural habitat conditions of *Chionodraco hamatus*. All ionizable residues were assigned their default protonation states at neutral pH (7.0). The resulting molecular system contained 192,380 atoms for PepT1-HS and 387,974 atoms for PepT1-CH. Computational simulations were conducted using NAMD3 [19] leveraging GPU acceleration technology for improved processing efficiency, employing the CHARMM36m [20] force field for protein components and CHARMM36 [21] for lipid molecules. Long-range electrostatic forces were computed via the particle mesh Ewald (PME) approach with a 1.2 nm cutoff distance [22]. An identical cutoff was implemented for van der Waals interactions, incorporating a switching function beginning at 1.0 nm. Hydrogen-containing covalent bonds were kept rigid using the SHAKE constraint algorithm, permitting a 2 fs integration timestep. Initial system optimization employed the steepest descent minimization method, succeeded by a staged equilibration procedure in both NVT and NPT statistical ensembles. Following energy optimization, the assemblies underwent NVT equilibration (0.25 ns) and subsequent NPT equilibration (4.6 ns) through gradual reduction of restraining forces on lipids and proteins, plus an additional 150 ns of unrestrained NPT equilibration. Production-phase simulations operated under NPT conditions, maintaining constant temperature at 273 K for 500 ns and at 323 K for an additional 500 ns per system using Langevin temperature control, totaling 1 μs per system. Pressure regulation at 1 bar was achieved through the Langevin piston pressure control method with semi-isotropic coupling [23]. Simulation visualization and data analysis were performed using VMD [24] and Chimera X 1.10 [25] software packages. Protein-peptide binding free energy calculations were performed using the Molecular Mechanics Generalized Born Surface Area (MM-GBSA) method to quantitatively assess protein-ligand interactions [26]. Representative conformations were extracted from the final 100 ns of equilibrated MD trajectories at 2 ns intervals, yielding 50 snapshots per system for analysis. MM-GBSA calculations were conducted using MolALCAL [27], which computed the binding free energy according to the equation ΔG_binding_ = ΔE_MM_ + ΔG_sol_, where ΔE_MM_ represents the molecular mechanics energy difference (electrostatic and van der Waals contributions) and ΔG_sol_ corresponds to the solvation free energy change. The Generalized Born implicit solvation model was employed with dielectric constants of 1.0 for the protein interior and 80.0 for the aqueous environment, incorporating a physiological salt concentration of 400 mM. Nonpolar solvation energies were calculated using the Linear Combination of Pairwise Overlaps (LCPO) method with a surface tension coefficient of 0.0072 kcal/(mol·Å^2^). For each trajectory frame, the total binding energy was determined by subtracting the individual energies of the isolated receptor and ligand from the energy of the protein–ligand complex. Final binding free energies were obtained by averaging over all selected conformations, with standard errors calculated to assess statistical convergence. Entropy contributions were omitted due to their high computational cost and limited impact on relative binding affinity rankings.

### 2.1. Lipid Interaction Analysis and Order Parameters

Lipid interaction analysis was performed using custom Tcl scripts in VMD. For lipid proximity, the minimum distance between each protein residue (Cα atom) and any POPC lipid atom was calculated in each frame and then averaged over the entire trajectory. These values were saved and mapped to the β-factor field of a new PDB file to enable visualization. For lipid occupancy, the percentage of simulation frames in which each residue was in contact with POPC lipids (within a 5.0 Å cutoff) was computed over the entire trajectory. Contact was counted when any atom of a given residue was within the threshold distance of any lipid atom. The frame-wise contact frequency was converted to a per-residue occupancy percentage, providing a time-averaged view of lipid interactions at the residue level. All analysis scripts used are provided as Supplementary Material.

The lipid order parameter, commonly denoted as S_CD_, is used to quantify the degree of order of the hydrocarbon chains in a lipid bilayer. It is especially relevant in molecular dynamics simulations and NMR studies. A widely used formula is [28]:

(1) $$S_{CD}=\langle \frac{{3cos}^{2}\Theta -1}{2}\rangle$$

where *θ* is the angle between the lipid chain axis (typically the average over all C-C bond vectors of the lipid-molecule tail) and the bilayer normal (usually the z-axis of the system) and ⟨·⟩ denotes an average over time and/or over multiple lipid molecules. The order parameter was calculated using a Tcl script in VMD.

### 2.2. Dynamic Cross-Correlation Analyses

To evaluate the effect of temperature on the protein dynamics, dynamical cross-correlation maps [29,30] were calculated using the Bio3D package in R [31]. Dynamic cross-correlation matrices with coefficients

*C*_*ij*_ = 〈Δr_i_·Δr_j_〉〈Δr_i2_〉〈Δr_j2_〉 (2)

were determined from the positions of the main chain Cα atoms in amino acids *i* and *j* with positions *r*_*i*_ and *r*_*j*_. Δ*r*_*i*_ and Δ*r*_*j*_ determine the displacement of the *i*th Cα from its mean position over the entire trajectory.

## 3. Results

### 3.1. Enhanced Global Flexibility and Temperature-Responsive Dynamics in the Cold-Adapted ChPepT1

**Figure 1:**
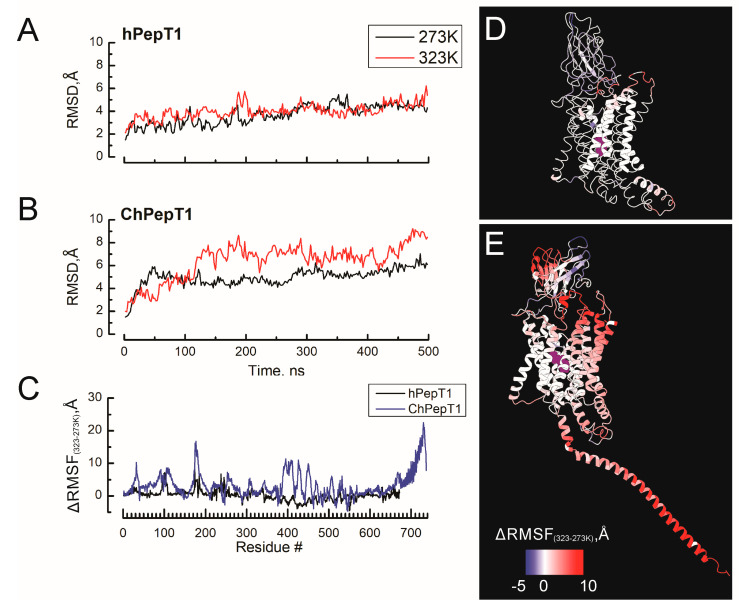
Temperature-dependent structural flexibility of hPepT1 and ChPepT1. (**A**,**B**) Root mean square deviation (RMSD) of human (hPepT1, (**A**)) and Antarctic icefish PepT1 (ChPepT1, (**B**)) over 500 ns of molecular dynamics at 273 K (black) and 323 K (red), showing greater conformational variability in ChPepT1. (**C**) Per-residue change in root mean square fluctuation (ΔRMSF = RMSF_323K_ − RMSF_273K_) for hPepT1 (black) and ChPepT1 (blue), highlighting regions with increased temperature sensitivity in the cold-adapted variant. Structural mapping of ΔRMSF values onto hPepT1 (**D**) and ChPepT1 (**E**). Color scale from blue (decreased flexibility at higher temperature) to red (increased flexibility at higher temperature). The dipeptide substrate is shown as purple spheres at the binding site.

The work by Rizzello et al. (2013) showed that the PepT1 of *Chionodraco hamatus* (ChPepT1) exhibited a remarkable cold tolerance, as its activity experiences significantly small change with temperature compared with those from a temperate species [16]. A common strategy for developing cold tolerance among soluble proteins of Antarctic organisms is their increased flexibility, which allows them to undergo the conformational changes required for their activity even in conditions where little kinetic energy is available [32]. To study the molecular features of the PepT1 that confer cold tolerance, we performed MS at 273 and 323 K of the human and Antarctic icefish variants of the PepT1 in the occluded/outward-facing state. We performed molecular simulations beginning at 273 K after a 100 ns equilibration protocol and remaining at this temperature during 500 ns. After that time, the temperature increased to 323 during the next 500 ns. To evaluate the overall protein stability, the average root mean square deviation (RMSD) of human and icefish PepT1 systems was calculated to assess protein stability throughout the simulation time. As shown in Figure 1A, human PepT1 was more stable and less responsive to temperature changes, with RMSD values of 3.7 ± 0.8 Å and 4 ± 0.6 Å at 273 K and 323 K, respectively. In contrast, icefish PepT1 showed larger deviations from the initial structure, which increased with temperature, reaching RMSD values of 4.69 ± 0.8 Å and 6.3 ± 1.5 Å at 273 K and 323 K, respectively (Figure 1B). This significant increase in structural deviation suggests that the icefish PepT1 variant undergoes large conformational changes at higher temperatures, which may be relevant to its adaptation to cold environments. To assess the existence of species-specific thermal tolerance hotspots accounting for the cold adapting phenotype, the average root mean square fluctuation (RMSF) was calculated across all MD simulations to evaluate the atomic mobility of individual residues. Higher RMSF values implied greater mobility, while lower RMSF values indicated restricted movements. Figure 1C displays the temperature-dependent change in the average root mean square fluctuation (∆RMSF_323–273K_) values at 273 K and 323 K for human and Antarctic PepT1, respectively, considering all the atoms of each residue, providing overall structural flexibility. The ∆RMSF_323–273K_ were mapped into the protein structure of hPepT1 (D) and ChPepT1 (E), where residues are color-coded according to scale bar. In line with previous studies of cold-adapted enzymes [33,34,35], our analysis reveals that the cold-adapted variant, ChPepT1, is intrinsically more flexible than hPepT1. This indicates a fundamental difference in the baseline structural dynamics between the two transporters. When comparing temperature-induced fluctuations, we observed that hPepT1 exhibited only a moderate increase in flexibility with rising temperature, primarily limited to TM3, the loop connecting TM5 and TM6, and the intracellular domain (ICD). In contrast, ChPepT1 showed a substantial increase in RMSF upon temperature elevation, indicating that its structural flexibility is more sensitive to thermal variation. Beyond the temperature-responsive regions also found in hPepT1, ChPepT1 displayed pronounced fluctuations in the extracellular domain (ECD) and the C-terminal domain (CTD). Similar to findings in soluble enzymes, [4,36] several of the temperature-sensitive regions in ChPepT1 are located within 5 Å of the peptide-binding site, supporting the idea that cold tolerance may arise from temperature-dependent modulation of substrate binding affinity.

### 3.2. Thermodynamic Buffering of Substrate Binding Affinity in ChPepT1 Across Temperatures

**Figure 2:**
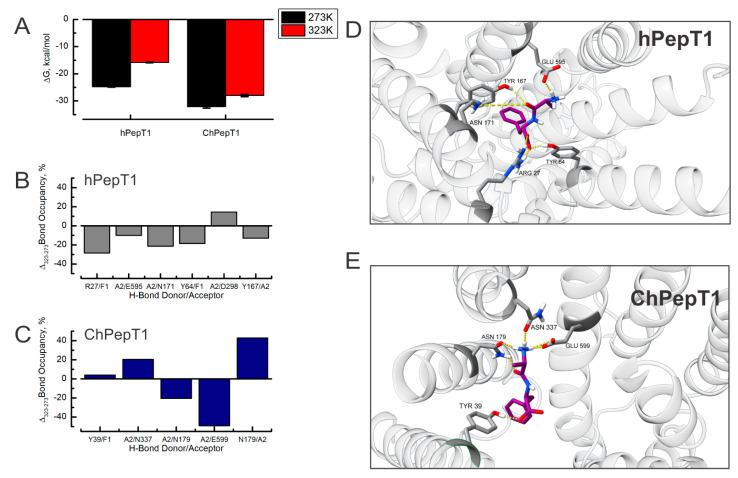
Cold-driven changes on hydrogen bonding and binding energetics in hPepT1 and ChPepT1 transporters. (**A**) Binding free energies (ΔG) of the substrate to hPepT1 and ChPepT1 at 273 K (black) and 323 K (red), showing temperature-dependent changes in substrate affinity. (**B**,**C**) Cold-induced differences in hydrogen bond occupancy (Δ% bond occupancy, 273–323 K) for specific residue pairs in hPepT1 (**B**) and ChPepT1 (**C**). Structural snapshots for the peptide binding site and the hydrogen bond network are depicted for the hPepT1 (**D**) and ChPepT1 (**E**).

The direct relationship between the transport rate and temperature in several membrane transporters arises from a combination of decrease in the substrate binding affinity and larger protein fluctuations. Functional assays showed that hPepT1 exhibits a 50% decrease in Km between 20 °C and 30 °C, while the transport currents increase threefold [37]. In contrast, ChPepT1 displays transport currents that are nearly temperature-independent [16], although its binding affinity temperature dependence has not yet been characterized. To address whether the ChPepT1 experiences temperature-dependent changes in its binding affinity for the substrate that may explain its lack of temperature dependence in their transport currents, we performed Molecular Mechanics/Generalized Born Surface Area (MM/GBSA) calculations to investigate potential species-specific differences in binding energetics. Our results show that hPepT1 exhibited a significant decrease in affinity with an increasing temperature, shifting from −24.7 kcal/mol at 273 K to −15.8 kcal/mol at 323 K. In contrast, ChPepT1 maintained a relatively stable ΔG, ranging from −32.1 kcal/mol to −27.9 kcal/mol across the same temperature range (Figure 2A). Table 1 shows the individual components of the Gibbs free energy of binding obtained from the MM-GBSA calculations. When the changes in ΔG are dissected into their individual components, we observe that in hPepT1, most of the temperature-dependent loss of binding affinity arises from a decrease in the electrostatic energy (ΔE_electrostatics_) and the solvation free energy (G_solvation_). In contrast, ChPepT1 exhibits a smaller temperature-dependent change in binding affinity, with the observed variation primarily attributable to a reduction in the van der Waals energy (ΔE_VDW_). These findings suggest that ChPepT1 retains strong substrate binding even at higher temperatures, whereas hPepT1 undergoes a marked loss of affinity as temperature increases. To understand the molecular basis of this difference, we analyzed the hydrogen bonding within the complex during simulations at both temperatures. To dissect this further, we quantified temperature-induced changes in hydrogen bond occupancy for each transporter. In hPepT1, all key hydrogen bonds between the peptide substrate and binding-site residues—such as R27, Y64, and E595—showed decreased occupancy at higher temperature (Figure 2B), correlating with the reduced binding affinity. In contrast, ChPepT1 demonstrates a thermodynamically buffered interaction profile: some hydrogen bonds decrease in occupancy (e.g., N179/F1), but others—especially involving E599 and N179—compensate with enhanced stability at low temperature (Figure 2C). The corresponding structural snapshots (Figure 2D,E) provide a representative view of the binding site and the hydrogen bonding interactions during the molecular simulations. These results support a model in which ChPepT1 retains strong substrate interactions through a temperature-dependent rearrangement of its hydrogen bond network, mitigating the destabilizing effects of thermal fluctuation.

### 3.3. Cold-Dependent Proteolipid Coupling and Membrane Flexibility in ChPepT1

**Figure 3:**
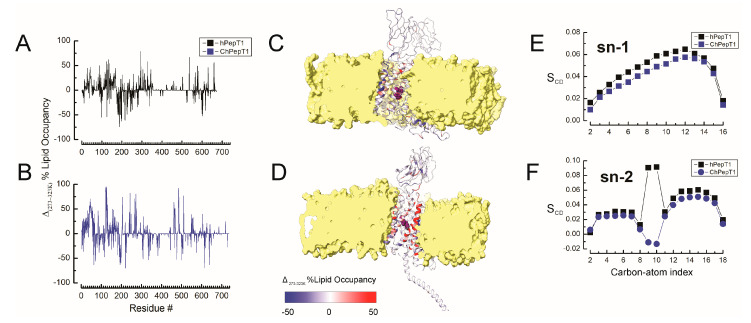
Temperature-dependent changes in protein/lipid interactions and membrane dynamics surrounding hPepT1 and ChPepT1. Temperature-dependent change in lipid occupancy (Δ% lipid occupancy 273–323 K) for (**A**) hPepT1 and (**B**) ChPepT1, showing residue-specific variation in lipid contacts between cold and warm conditions. Structural representations of hPepT1 (**C**) and ChPepT1 (**D**) embedded in a lipid bilayer. Protein cartoons are color-coded by lipid contact variation (Δ% lipid occupancy 273–323 K), with red indicating increased lipid contact at low temperature and blue indicating decreased contact. Lipid density is shown as a yellow isosurface. Deuterium order parameters (S_CD_) for sn-1 (**E**) and sn-2 (**F**) acyl chains of POPC lipids near hPepT1 (black) and ChPepT1 (blue), reflecting differences in local membrane fluidity. Lower S_CD_ values near ChPepT1 indicate enhanced lipid disorder consistent with cold adaptation.

The lipid environment surrounding membrane proteins plays a critical role in modulating protein flexibility and is therefore expected to influence cold tolerance in psychrophilic proteins [38,39,40]. To better understand the cold adaptation of the icefish proton-coupled peptide transporter ChPepT1, we analyzed the percentage of simulation frames in which each protein residue was within 5 Å of any POPC lipid (script provided in the Supplementary Material). To assess how the lipid environment contributes to cold adaptation in PepT1 transporters, we analyzed per-residue lipid occupancy and cold-dependent changes in lipid chain order (ΔS_CD_) at 5 Å of both hPepT1 and ChPepT1 (Figure 3A–E). Lipid occupancy analysis (Figure 3A,B) revealed that ChPepT1 shows increased lipid contact at 273 K compared to 323 K, particularly in regions near the C-terminal domain and transmembrane helices adjacent to the substrate-binding site. This redistribution suggests that ChPepT1 leverages enhanced proteolipid interactions to stabilize flexible regions under cold conditions. Structural maps confirm a greater extent of protein–lipid contact in ChPepT1 at lower temperatures (Figure 3C,D). To evaluate membrane physical properties, we calculated the deuterium order parameter (S_CD_) for the sn-1 and sn-2 acyl chains of POPC lipids surrounding each transporter. The S_CD_ (Figure 3E,F), defined as the difference between 323 K and 273 K, shows a greater loss of order in lipids near ChPepT1, reflecting a more dynamic membrane environment. As expected, increasing temperature led to greater lipid disorder in both systems [41,42]; however, lipids surrounding ChPepT1 maintained higher disorder overall, especially in the unsaturated region. In line with the homeoviscous adaptation model [43], these findings support a model in which ChPepT1’s cold adaptation involves both structural flexibility and enhanced interaction with a more fluid, dynamically rearranged lipid environment. This interplay likely helps maintain transporter activity in low-temperature conditions by providing entropic buffering through the proteolipid interface.

### 3.4. Temperature-Induced Structural Remodeling and Membrane Decoupling of the C-Terminal Domain in ChPepT1

The C-terminal domain (CTD) of ChPepT1 has been identified as a critical determinant of cold adaptation. Rizzello et al. [16] showed that a single heptad repeat (VDMSRKS) within this domain is sufficient to confer cold tolerance when introduced into a temperate ortholog, highlighting its functional significance. Interestingly, ChPepT1 possesses a significantly longer CTD than hPepT1, suggesting that additional structural elements may contribute to its adaptive behavior under thermal stress.

**Figure 4:**
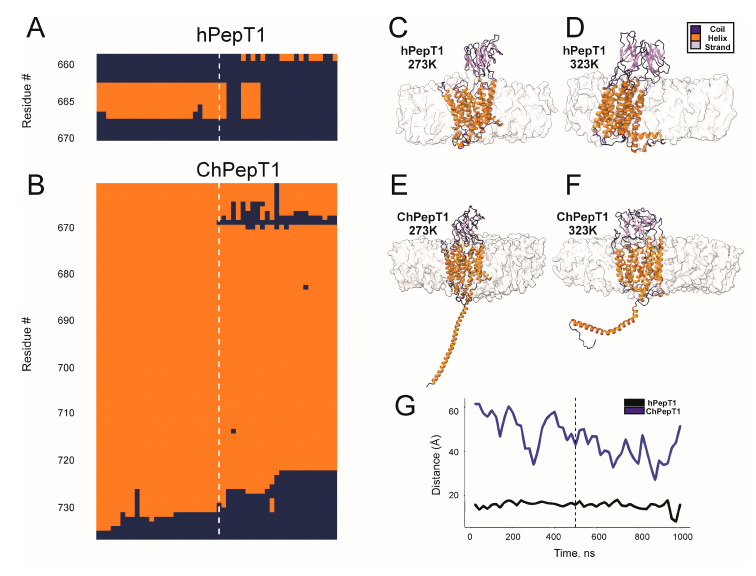
Temperature-dependent secondary structure changes and spatial displacement of the CTD in hPepT1 and ChPepT1. (**A**,**B**) Secondary structure assignment over time for the proximal CTD in hPepT1 (**A**) and ChPepT1 (**B**) during molecular dynamics simulations at 273 K (left of dashed line) and 323 K (right of dashed line). Residues are colored by secondary structure: orange indicates α-helix, and dark blue indicates random coil. (**C**–**F**) Representative snapshots of the CTD conformation and spatial orientation in hPepT1 (**C**,**D**) and ChPepT1 (**E**,**F**) at 273 K and 323 K, respectively. α-helix are shown in orange, random coils in blue, and membrane density as a transparent surface. At 273 K, the CTD in ChPepT1 remains anchored to the intracellular face of the membrane, while at 323 K it exhibits marked displacement and extension away from the bilayer. (**G**) Distance between the CTD and the membrane center over time in hPepT1 (red) and ChPepT1 (blue). Dashed line indicates the temperature switch from 273 K to 323 K.

Figure 4 illustrates temperature-dependent conformational remodeling in this region. In both hPepT1 and ChPepT1, residues 660–670 at the proximal end of the CTD undergo an α-helix to coil transition upon heating, a region we refer to as the temperature-responsive segment (TRS) (Figure 4A,B). In ChPepT1, however, this transition extends into the distal CTD, implying a more extensive structural rearrangement in response to temperature changes. Representative structural snapshots at 273 K and 323 K (Figure 4C–F) show clear differences in CTD conformation and its spatial relationship to the membrane. Figure 4G quantifies the displacement of the CTD center of mass relative to the membrane. In hPepT1, the CTD remains stably anchored throughout the simulations. By contrast, ChPepT1 shows a pronounced translational shift of over 20 Å, consistent with a temperature-dependent membrane detachment. This dynamic decoupling may function as a thermal insulation mechanism, allowing the transporter to maintain its conformational flexibility and activity under cold conditions.

### 3.5. Temperature-Modulated Interdomain Coupling Reveals Dynamic Network Reorganization in ChPepT1

**Figure 5:**
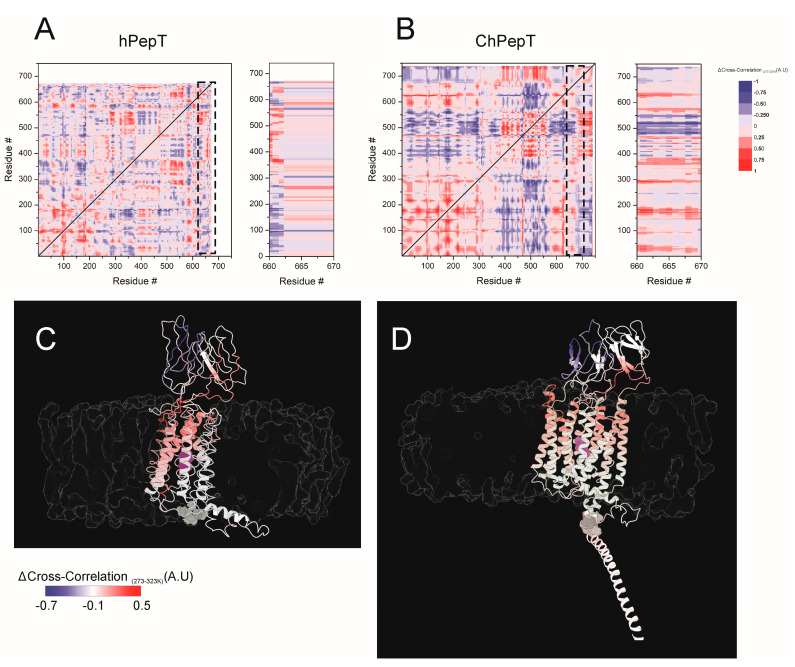
Cold-driven reorganization of dynamic coupling in hPepT1 and ChPepT1. (**A**,**B**) Temperature-dependent changes in the cross-correlation matrices of residue–residue motions between 273 and 323 K for hPepT1 (**A**) and ChPepT1 (**B**), calculated from MD trajectories. Right insets highlight interactions involving residues 660–670 (Temperature responsive segment, TRS), revealing distinct coupling patterns across the protein. ChPepT1 shows stronger correlations between the TRS and transmembrane domains, indicating enhanced dynamic integration. (**C**,**D**) Structural mapping of Δ Cross-correlation (323–273 K) in hPepT1 (**C**) and ChPepT1 (**D**). Residues are colored by the degree of temperature-induced correlation change, from blue (decreased coupling) to red (increased coupling). ChPepT1 exhibits temperature-sensitive reorganization of domain interactions, including strengthened coupling between the CTD and substrate-binding transmembrane helices, absent in hPepT1. The dipeptide ligand is shown in purple; the TRS (660–670) is shown in grey and lipid bilayer is depicted as a transparent surface.

Dynamic cross-correlation (DCC) analysis is a widely used approach to investigate the cooperative and antagonistic motions between different regions of a protein during molecular dynamics simulations, offering valuable insight into conformational plasticity and allosteric communication [44,45,46]. In the context of membrane transporters, such as PepT1, DCCM provides a powerful framework to explore how domain interactions are modulated under varying environmental conditions, including temperature shifts. To address how temperature modulates interdomain dynamic coupling in PepT1 transporters, we analyzed differential cross-correlation maps (ΔCC = CC_273K_ − CC_323K_) for hPepT1 and ChPepT1 (Figure 5A,B, left). In hPepT1, cold-induced changes in dynamic correlation are minimal, with ΔCC values remaining within a narrow range (−0.25 to 0.25), indicating limited sensitivity of the protein’s dynamic architecture to cold exposure. The insets in Figure 5A,B (right) highlight the temperature-dependent changes in coupling between TRS and the rest of the protein (see also Figure 4). In hPepT1, TRS shows only modest shifts in correlation with the rest of the protein, suggesting that despite its local structural transition, it remains functionally isolated and does not strongly influence the dynamic behavior of other domains. In stark contrast, ChPepT1 displays extensive temperature-dependent reorganization of dynamic coupling. Significant increases in correlation (ΔCC > 0.5) are observed in key transmembrane regions, including residues 25–50 (TM1), 160–200 (TM5), 280–295 (TM7), and 615–630 (TM11). Concurrently, a marked decoupling (ΔCC < −0.5) is seen between the extracellular domain (residues 475–525) and the proximal CTD, indicating that both domains dynamically disengage at lower temperatures. Taken together, these results suggest that while hPepT1 exhibits a static and weakly connected CTD that fails to engage with other functional domains while ChPepT1 actively reorganizes its dynamic network in response to cold, functionally coupling the CTD more tightly to the transmembrane region and potentially preserving transport activity under low-temperature conditions.

## 4. Discussion

Cold environments place significant constraints on protein function by limiting the thermal energy required for conformational transitions. These limitations are especially severe for membrane proteins, where both intramolecular flexibility and membrane viscosity must be balanced to sustain catalytic activity. While cold adaptation in soluble enzymes is often attributed to a small number of mutations that reduce conformational enthalpy and enhance flexibility [5,7,47], our findings show that similar principles apply to membrane transporters, but through multi-layered and distributed mechanisms. Our molecular simulations revealed that the Antarctic peptide transporter ChPepT1 is substantially more flexible than its human ortholog, particularly in regions adjacent to the substrate-binding pocket (Figure 1). This global elevation in baseline flexibility likely represents a compensatory adaptation that facilitates essential conformational changes despite limited thermal energy. Notably, the extracellular domain (ECD) and the carboxy-terminal domain (CTD) displayed high thermal sensitivity and fluctuation amplitudes, consistent with their potential role in modulating substrate access and transport rate at low temperatures [16,48].

In parallel, binding free energy analyses (Figure 2) showed that substrate affinity in ChPepT1 remains remarkably stable between 273 K and 323 K, in contrast to the steep temperature-dependent decline observed in hPepT1. This thermodynamic buffering likely arises from a reorganization of noncovalent interactions, where weakened hydrogen bonds at low temperatures are compensated by the formation of new, transient interactions. Such mechanisms resemble those observed in psychrophilic enzymes that reduce activation barriers (ΔG^‡^) by disrupting overly rigid interaction networks and enhancing local plasticity [3,49]. Beyond structural properties, our lipid interaction analysis (Figure 3) underscores the role of the membrane environment in thermal adaptation. ChPepT1 exhibited enhanced lipid occupancy near specific transmembrane helices (e.g., TM1–2, TM4) and consistently promoted higher lipid disorder, even under cold conditions. These findings support the homeoviscous adaptation hypothesis [43] in which membrane fluidity is maintained via protein–lipid interactions that are fine-tuned to promote conformational freedom. The observation that ChPepT1 maintains a more disordered proteolipid interface underlies a novel layer of adaptation, in which membrane components dynamically support the structural requirements of cold-adapted transport. The work by Rizzello et al. demonstrated that a single heptad repeat (VDMSRKS) within the C-terminal domain (CTD) is sufficient to confer the cold-adaptive phenotype to rabbit PepT1 [16]. Interestingly, a distinctive feature of ChPepT1 is that the temperature-responsive segment (TRS) encompassing residues 660–670 located immediately upstream of the heptad is exposed to the cytosol rather than embedded in the lipid bilayer, as seen in hPepT1 (Figure 4). In this context, it is worth noting that the highly polar character of the heptad could facilitate or stabilize the displacement of the adjacent TRS away from the membrane. This spatial reorientation may be sufficient to enable the TRS to adopt a cold-tolerant conformation, suggesting that even a single heptad could indirectly promote cold adaptation by altering the positioning of the nearby TRS. Dynamic cross-correlation analyses (Figure 5) revealed that cold induces a temperature-dependent reorganization of interdomain communication. In hPepT1, long-range correlations remain largely static across temperatures. In contrast, ChPepT1 undergoes a rewiring of long-range communication, characterized by weakened global contacts (e.g., ECD–TM1–4) and enhanced local communication within TM9 and TM11—regions essential for substrate gating [17]. These changes reflect a shift from global coordination to localized control, a strategy that allows core transport motions to persist even under reduced thermal energy conditions. This perspective aligns with previous work on cold-adapted enzymes, where dynamic allostery—rather than large-scale structural changes—was shown to underlie cold tolerance. For example, Saavedra et al. demonstrated that local unfolding in distal, non-active regions can entropically modulate substrate affinity and catalytic turnover, allowing function to persist at low temperature despite a largely conserved structural fold [50]. Although their work focused on soluble enzymes, the core principle of entropic tuning through domain-specific flexibility may also apply to membrane transporters like ChPepT1. In our case, the temperature-dependent reorganization of interdomain communication, combined with enhanced local flexibility near the substrate-binding site and within transmembrane helices, likely represent a comparable mechanism, allowing function to be preserved in the cold through distributed dynamic adaptation rather than structural reengineering. Similar dynamic remapping has been observed in other transporters where conformational gating, such as occlusion and deocclusion, is the rate-limiting step [51,52]. Together, these results support a model in which cold tolerance in ChPepT1 arises from intrinsic flexibility, thermodynamically buffered substrate interactions, lipid-tuned transmembrane dynamics, and selectively rewired interdomain communication. These adaptations parallel those reported for other cold-adapted membrane proteins, such as the Na^+^/K^+^-ATPase from Antarctic octopus, where a small number of lipid-facing mutations were sufficient to confer cold tolerance by modulating protein–lipid coupling and local flexibility [14]. Moving forward, site-directed mutagenesis targeting flexible loops and lipid-facing helices—particularly within the CTD and ECD, combined with electrophysiological validation, will be essential to establish causal links between structure, dynamics, and cold tolerance.

## 5. Conclusions

In conclusion, our findings suggest that cold adaptation in the Antarctic peptide transporter ChPepT1 involves a distributed and multifaceted strategy. This includes elevated baseline flexibility, thermodynamically buffered substrate binding, membrane lipid interactions that promote fluidity, and temperature-dependent reorganization of interdomain communication. These adaptations enable essential conformational transitions under low thermal energy conditions and parallel mechanisms observed in other psychrophilic membrane proteins. While the precise causal relationships remain to be fully defined, this study underscores the complexity of cold adaptation in membrane transporters and lays a foundation for future functional validation through targeted mutagenesis.

## Figures and Tables

**Table 1:** Temperature-dependent binding free energy components for hPepT1 and ChPepT1.

	hPepT1 (kcal/mol)		chPepT1 (kcal/mol)	
Energy Component	273 K	323 K	273 K	323 K
∆E (Internal)	0	0	0	0
∆E (Electrostatic) + ∆G(Solvation)	−9.3	0.23	−15.9	−16.05
∆E (VDW)	−15.4	−16.06	−16.17	−11.9
G (Binding)	−24.7 ± 0.14	−15.8 ± 0.16	−32 ± 0.52	−27.9 ± 0.46

MM-GBSA calculations of dipeptide binding to human (hPepT1) and Antarctic icefish (ChPepT1) PepT1 at 273 K and 323 K. The binding free energy (ΔG) is decomposed into electrostatic plus solvation contributions and van der Waals (VDW) interactions. While hPepT1 shows a marked loss of binding affinity at higher temperature, ChPepT1 maintains a stable and favorable binding profile across temperatures. Values are reported in kcal/mol as means ± standard error from 50 simulation snapshots.

## Data Availability

The original contributions presented in this study are included in the article/Supplementary Material. Further inquiries can be directed to the corresponding author.

## Supplementary Materials

The following supporting information can be downloaded at: https://www.mdpi.com/article/10.3390/biom15081058/s1, File S1: The Tcl scripts for lipid analysis shown in Figure 3.

## Abbreviations

The following abbreviations are used in this manuscript:

ChPepT1	*Chionodraco hamatus* peptide transporter 1
hPepT1	Human peptide transporter 1
CTD	C-terminal domain
ECD	Extracellular domain
TRS	Temperature-responsive segment
TM	Transmembrane (e.g., TM1, TM5)
MM/GBSA	Molecular Mechanics/Generalized Born Surface Area
RMSD	Root Mean Square Deviation
RMSF	Root Mean Square Fluctuation
MD	Molecular Dynamics
DCC	Dynamic Cross-Correlation
DCCM	Dynamic Cross-Correlation Matrix
S_CD_	Lipid order parameter (deuterium order parameter)
PME	Particle Mesh Ewald
LCPO	Linear Combination of Pairwise Overlaps
POPC	Palmitoyl-oleoyl-phosphatidylcholine
TIP3P	Transferable Intermolecular Potential with 3 Points
CHARMM-GUI	Chemistry at HARvard Macromolecular Mechanics—Graphical User Interface
VMD	Visual Molecular Dynamics
PDB	Protein Data Bank
NPT	Constant Number, Pressure, Temperature ensemble
NVT	Constant Number, Volume, Temperature ensemble
hASA	Hydrophobic Accessible Surface Area
Ea	Activation energy
ΔG	Gibbs free energy
ΔG‡	Activation free energy barrier
ΔCC	Change in cross-correlation
ICD	Intracellular domain
pLLDT	Predicted Local Distance Difference Test (confidence score from AlphaFold)
